# Dose-Response Effects of MittEcho, a Measurement Feedback System, in an Indicated Mental Health Intervention for Children in Municipal and School Services in Norway

**DOI:** 10.1007/s10488-024-01389-9

**Published:** 2024-05-29

**Authors:** Ida Mari Haug, Simon-Peter Neumer, Bjørn Helge Handegård, Carina Lisøy, Lene-Mari P. Rasmussen, Elisabeth Valmyr Bania, Frode Adolfsen, Joshua Patras

**Affiliations:** 1 Regional Centre for Child and Adolescent Mental Health and Child Welfare, Northern Norway, UiT The Arctic University of Norway, Sykehusvegen 44, Tromsø, 9019 Norway; 2 Regional Centre for Child and Adolescent Mental Health, Eastern and Southern Norway, Oslo, Norway; 3 Regional Centre for Child and Youth Mental Health and Child Welfare, Department of Mental Health, Central Norway, Norwegian University of Science and Technology, Trondheim, Norway

**Keywords:** Measurement feedback system (MFS), Routine outcome monitoring (ROM), School mental health services, Children & young people, Group intervention, Cognitive behavioral therapy (CBT)

## Abstract

Including routine client feedback can increase the effectiveness of mental health interventions for children, especially when implemented as intended. Rate of implementation, or dose, of such feedback interventions has been shown to moderate results in some studies. Variation in implementation and use of client feedback may also contribute to the mixed results observed within the feedback literature. This study evaluates dose-response associations of client feedback using a novel Measurement Feedback System (MFS) within an indicated group intervention. The primary aim was to determine whether the rate of MFS implementation predicts symptom reduction in anxiety and depression among school-aged children. The secondary aim was to assess whether the rate of MFS implementation influences children’s satisfaction with the group intervention or their dropout rates. Data were collected via a randomized factorial study (clinicaltrials.gov NCT04263558) across 58 primary schools in Norway. Children aged 8 to 12 years (*N* = 701) participated in a group-based, transdiagnostic intervention targeting elevated symptoms of anxiety or depression. Half of the child groups also received the feedback intervention using the MittEcho MFS. Group leaders (*N* = 83), recruited locally, facilitated the interventions. The MFS dose was measured using the Implementation Index, which combines the use of MFS by both children and providers (group leaders) into a single dose variable. Results showed no significant additional effect of dose of MFS on change in depression or anxiety scores, on user satisfaction with the intervention or on intervention dropout. The discussion addresses potential reasons for these non-significant findings and implications for MFS implementation in preventive, group-based interventions in school settings.

**Supplementary Information:**

The online version contains supplementary material available at 10.1007/s10488-024-01389-9.

Routine collection and use of client outcome data has the potential to improve intervention outcomes among children and adolescents in community mental health settings (Rognstad et al., 2023). However, there is a notable gap in research focusing on children and adolescents, especially in indicated or group-based settings (de Jong et al., 2021). Furthermore, studies examining the effectiveness of routine use of client outcome data for youth have reported mixed results (Bergman et al., 2018; Rognstad et al., 2023; Tam & Ronan, 2017). It remains unclear whether the mixed results are attributable to the youth population, the heterogeneous intervention formats, or the treatment settings. Another possible explanation can be that adherence to feedback interventions is low. The practice has proven challenging to implement, and various barriers have been identified (Mackrill & Sørensen, 2019). Suboptimal utilization likely limits the potential for positive effects of client feedback. Studies have demonstrated that the level of implementation as measured by a combination of respondents´ response-rates and therapists viewing-rates, known as the *Implementation Index*, significantly impact the effect of the use of client outcome data (Bickman et al., 2016).

The practice of routinely collecting client data and monitoring progress to inform treatment decision are referred to by several terms that are often used interchangeably in the literature. We believe the terms feedback intervention or routine outcome monitoring (ROM) best characterize the practice described in this article (Lambert & Harmon, 2018). Measurement feedback systems (MFS) are digital tools designed to facilitate the collection and display of user feedback, and supports the ROM practice (Bickman, 2008). While standardized measures on outcomes such as symptoms of depression and anxiety are most used in ROM and MFS, the use of idiographic, or client generated aims and outcomes, has gained popularity (Lloyd et al., 2019; Lyon et al., 2016; Rognstad et al., 2023; Sales & Alves, 2016).

This client-centered practice is believed to enhance intervention effectiveness by identifying individuals who are not improving as expected, enabling therapists to adjust the intervention to better meet an individual’s needs (Howard et al., 1996). Research with idiographic measures also indicates that goal setting may facilitate behavior change and reduce external attributions (Epton et al., 2017; Tollefsen et al., 2020). The mechanisms through which ROM work may include at least three core components: (1) the routine collection of client data, (2) the provision of this data to the therapist as feedback, and (3) the integration of feedback into clinical decision making and adaptation of the intervention, often in collaboration with the client (Barber & Resnick, 2022; Barkham et al., 2023; Lewis et al., 2019).

There is evidence that adding ROM using MFS to usual practice in mental health services has several benefits, although the added effects are small (*d* = 0.11–0.15; de Jong et al., 2021; Rognstad et al., 2023). These benefits include greater and faster symptom reduction, increased functioning and life satisfaction, higher treatment satisfaction, reduced dropout, and the fostering of therapeutic alliance and collaboration (de Jong et al., 2021; Gondek et al., 2016; Lambert et al., 2018; Rognstad et al., 2023). ROM seems to particularly benefit those at risk of worsening or not improving, known as “not on track” cases (NOT; Lambert, 2003). Research with idiographic measures indicates that goal setting may facilitate behavior change and reduce external attributions (Epton et al., 2017; Tollefsen et al., 2020).

Studies on the effect of feedback have predominantly focused on adults in counselling settings and one-to-one therapy (de Jong et al., 2021). Yet approximately every fourth and fifth child and young person experiences elevated symptoms of anxiety or depression, and mental health problems are among the largest causes contributing to the global burden of disease (GBD 2019Viewpoint Collaborators, 2020; Racine et al., 2021). Heightened symptoms in childhood pose a significant risk of a psychiatric diagnosis later in life and comorbid diagnoses (Caspi et al., 2020; Mulraney et al., 2021). Timely and targeted prevention efforts can mitigate processes leading to later mental illness (Colizzi et al., 2020; Conley et al., 2017). Research also indicates that ROM and MFS can be more effective in outpatient and counseling settings than inpatient settings, indicating that there may be a potential of MFS also in preventive interventions (Davidson et al., 2015; de Jong et al., 2018; Østergård et al., 2020).

Though there is less research including children and adolescents, the available evidence suggests benefits for this group as well (Rognstad et al., 2023; Tam & Ronan, 2017). However, the evidence is not entirely conclusive (Bergman et al., 2018). Effectiveness studies with children and youth in community mental health services have often not been Randomized Controlled Trials (RCT) or have suffered from small sample sizes, which limits the generalizability and validity of results. In one of the few randomized trials, Bickman et al. (2011) conducted a study including 28 sites and 383 youths in a community counseling setting. They observed significant effects of feedback on symptom severity and function, with the feedback groups improving significantly faster than control groups as reported by the youths and clinicians. Research on the effects of feedback in low-threshold services, such as those provided in the school setting remains scarce. Though several authors discuss implementation of ROM in the school context (Borntrager & Lyon, 2015; Connors et al., 2015), and a review found 17 applicable studies with MFS and MFS-instruments that were appropriate for school age children (Dart et al., 2019). Two studies evaluating feedback in school mental health services in the UK have reported promising results. In the first, Cooper et al. (2013) found reduced within-group levels of distress among a cohort of children aged 7–11 years referred for social, emotional, or behavior problems. However, the absence of a control condition limited the validity of these results. In a second study, Cooper et al. (2021) conducted a cluster-randomized pilot in the same age group and in a school-based counseling setting. They found small to moderate effects of feedback; however, because of the small sample size (*n* = 38), generalizations are difficult to make.

Research on the efficacy of ROM in group therapy is also limited; only 10% of studies on feedback are conducted in group settings (de Jong et al., 2021). Most of these studies include adults and they show mixed results (e.g. Burlingame et al., 2018; Davies et al., 2008; Slone et al., 2015). However, there are indications that ROM may be highly appropriate in group-format. For example, there is evidence that NOT cases are more common in group settings compared to individual therapy (Alldredge et al., 2020), cases that often benefit the most from ROM (de Jong et al., 2021). Furthermore, the *Task Force of the American Group Psychotherapy Association* has recommended inclusion of processes and outcome measures in group therapy (Strauss et al., 2008). Less is known about the effects of ROM in group-based mental health interventions for children or adolescents, as only a few studies exist. Two studies investigated feedback in group therapy for children and youth aged 10 to 18 years in school counseling services (Shechtman & Sarig, 2016; Shechtman & Tutian, 2017). However, neither of these studies found positive effects of feedback on internalizing or externalizing problems.

The use and uptake of ROM and MFS is challenging, and various barriers to implementation have been identified in the literature (Gleacher et al., 2016; Lewis et al., 2019; Mackrill & Sørensen, 2019). Poor implementation and low fidelity are associated with reduced intervention effectiveness in general (Durlak & DuPre, 2008). Moreover, there are indications that the level of MFS utilization is connected to effects on mental health outcomes in therapy. For example, Bickman et al. (2011) found dose-response effects related to the number of feedback reports therapists viewed, with youth experiencing doubled effects when therapists viewed a higher proportion of reports. In a later RCT including two outpatient clinics for youth (age 11 to 18), the level of MFS implementation and its impact was assessed by combining information of respondent use and therapist viewing of the results, known as the Implementation Index (Bickman et al., 2016). The Implementation Index combines the rate of completed MFS measures from the respondents, and the rate at which therapists view these reports, resulting in a score ranging from 0 to 100. A score of 0 represents implementation failure and no dosage of MFS, whereas a score of 100 indicates complete implementation and maximum dose of MFS. Although the index does not measure actions taken based on feedback, it provides insight into the level of usage for both user groups, both of which are pre-requisites for MFS implementation. Using the index, Bickman et al. (2016) found evidence of dose-response effects for children and adolescents in one of the sites where faster improvement was related to greater MFS use. In the second site, no dose-response effect was significant, however, the implementation rate was much lower. Sale et al. (2021) also investigated MFS utilization with the Implementation Index, using naturalistic, archival data from a community mental health clinic serving children and youth. Higher scores on the Implementation Index predicted faster improvements for the children based on the caregiver reports.

Several other factors may also moderate the implementation of ROM, including characteristics of the child or the therapist (Johns et al., 2019; Karver et al., 2006; Sichel & Connors, 2022). Studies have shown that therapists with higher MFS implementation tend to experience fewer barriers and more facilitators to implementation than the low implementors, although the types of facilitators were not that different among them (Sichel & Connors, 2022). While therapist training and supervision or consultation are facilitators, they primarily enhance the knowledge of ROM and feedback collection, with less success in changing therapists’ attitudes and feedback utilization in treatment planning (Lyon et al., 2022). However, others do find that consultation predicts ROM fidelity (Woodard et al., 2023). Feedback utilization seems difficult for therapists and studies have demonstrated that this phase of feedback is the least common for therapists to engage in (Casline et al., 2022; Kwan et al., 2021). Therapists having favorable attitudes towards ROM also report that they use it more and attitudes were associated with greater effects on outcome (Rye et al., 2019). Further, therapist years of experience has been associated with both attitudes towards ROM and degree of implementation, where younger and inexperienced therapists are more likely to implement MFS (Connors et al., 2015; Kwan et al., 2021; Rye et al., 2019; Sale et al., 2020). However, the role of therapists’ experience in implementation or intervention effects are less clear (Johns et al., 2019; Sanchez-Bahillo et al., 2014). Others find no association for experience, ROM use, and outcome; yet therapists’ tendency to seek external information versus relying on their own perception seems important for utilization (de Jong et al., 2012). There are also youth characteristics that may predict ROM use. Children and youth may have greater benefit from ROM compared to adults, and older children and adolescents seem more likely to use it more and experience improvements of ROM (Rognstad et al., 2023; Smith & Jensen-Doss, 2017). However, few studies include children younger than 10 or 11 years, and if they do, it is rare for young children to self-report on their own outcomes (Tam & Ronan, 2017). Sex differences are rarely reported, yet many studies include female university students, which indicates benefits for females (Davidson et al., 2015). A qualitative study with group leaders from the same sample as the present study indicated that girls might have used the MFS more than boys (Haug et al., 2024). While severe intake or baseline symptoms may be connected to limited utility of MFS, some studies in community settings have shown higher effect for youth with higher initial severity (Davidson et al., 2015; Østergård et al., 2020; Smith & Jensen-Doss, 2017).

The present study investigated the use of MFS in an indicated, group-based mental health intervention for children. More specifically, we aimed to test whether a new MFS, and the degree to which it was used, would moderate children’s intervention outcomes. Lisøy et al. (2024) examined main effects of the MFS and the two other optimizing factors of the Emotion intervention on symptom reduction of anxiety and depressive symptoms. There were no significant main effects of the MFS, the other factors, or interactions between them. However, the study did not consider the degree of MFS utilization. As seen from previous research, actual use of MFS is a significant predictor for outcomes, and suboptimal use of MFS may have played a role in the null-findings in Lisøy et al. (2024). While no significant interactions between the MFS and the two other factors were found, it is possible that these factors interact with the MFS dosage. Parents in the high parental involvement conditions might have followed up on children’s MFS use more closely. The intervention format where half of the sessions were web-based might have put strain on children with too many out-of-session tasks to perform on their own; and it would also reduce group leaders’ opportunity to discuss feedback with children. Therefore, the primary aim of this study was to investigate whether the level of MFS use, measured by the Implementation Index, could predict reductions in anxiety and depressive symptoms. A secondary aim was to investigate hypotheses that MFS use would be associated with higher satisfaction with the primary intervention among the children, and that MFS would be associated with less intervention dropout.

The hypotheses were as follows:

i. Higher levels of MFS use, as measured by the Implementation Index, should be associated with greater reductions in children’s symptoms of anxiety.
ii. Higher levels of MFS use, as measured by the Implementation Index, should be associated with greater reduction in children’s symptoms of depression.
iii. Higher levels of use, as measured by the Implementation Index, should be associated with greater child satisfaction with the primary intervention.
iv. There should be less dropout from the intervention/study in the MFS condition compared to no-MFS condition.

## Methods

### Design & procedure

Data used in the current investigation was collected via the Echo-study (Clinicaltrials.gov NCT04263558), and the study was approved by the Regional Committees for Medical and Health Research Ethics (REK Southeast 28761) and The Norwegian Agency for Shared Services in Education and Research (Sikt; 152745). The data were collected during the fall of 2020 to spring 2022, with five cohorts (Neumer et al., 2021). The Echo-study is a national, factorial, cluster-randomized trial (*N* = 701 children) testing three factors for optimization of Emotion. Emotion is a transdiagnostic intervention based on principles of Cognitive Behavioral Theory (CBT) that targets elevated symptoms of depression and anxiety in young people (Martinsen et al., 2017a, b). The intervention is indicated, follows a manual, and is delivered in a group format with a maximum of seven children per group. Emotion has been found to be effective for reducing symptoms of anxiety and depression in a previous study (Martinsen et al., 2019). The Echo-study utilized a shorter version of Emotion to test a 2 × 2 × 2 factorial design with three factors: (1) delivery format, a 16 group-session arm vs. an arm with 8 web-based sessions and 8 group sessions, (2) parental involvement, with parents either receiving an information brochure or participating in dedicated parent sessions, and (3) MFS, weekly use of MFS vs. no MFS (Neumer et al., 2021). The combination of these three factors resulted in eight unique conditions, in which half of the sites were randomized to conditions with MFS. The current article focuses on the MFS-factor in this study and more precisely on the level of MFS use. The recruitment and the intervention took place locally in schools, and children were screened for symptoms of anxiety and depression and those scoring one standard deviation above the norm were invited to participate in the study. The group leaders in the study were provided with a two-day training and ongoing supervision in Emotion and the factors of the condition they were in. More details on the Echo-study’s design and procedures can be found in the study protocol (Neumer et al., 2021), or the publication of the post-intervention results from intent to treat analysis (Lisøy et al., 2024).

### Participants

The participants were 701 children and 83 group leaders recruited from different geographical regions of Norway. The children’s ages ranged from 8 to 12 years, and they were recruited from 4th, 5th, and 6th grade. After initial screening, the ones who scored one standard deviation above the expected mean for either, or both, depressive and anxious symptoms were invited to the group intervention. For symptoms of anxiety, a higher cutoff threshold was used for girls. There were 124 groups with an average of 6 children per group. Table 1 contains descriptive information for the full sample of children and for the MFS and no-MFS conditions. Forty-five children dropped out of the study and 22 had missing replies on the post-intervention survey.

**Table 1:** Sample characteristics for children in the full sample, MFS and No-MFS conditions

	MFS		No-MFS		Full sample	
	*n* (%)	M (SD)	*n* (%)	M (SD)	*n* (%)	M (SD)
*Sex of child*						
Girls	211 (58.8)		209 (61.1)		420 (59.9)	
Boys	148 (41.2)		133 (38.9)		281 (40.1)	
*Grade*						
4th	53 (14.8)		73 (21.3)		126 (18.0)	
5th	196 (54.6)		175 (51.2)		371 (52.9)	
6th	110 (30.6)		94 (27.5)		204 (29.1)	
*Reason for inclusion*						
Anxiety symptoms	291 (81.1)		281 (82.2)		572 (81.6)	
Depression symptoms	295 (82.2)		296 (86.5)		591 (84.3)	
Both	227 (63.2)		235 (68.7)		462 (65.9)	
Age		10.62 (0.70)		10.53 (0.68)		10.58 (0.69)
Baseline score anxiety		69.36 (15.16)		69.76 (14.45)		69.56 (14.81)
Baseline score depression		11.65 (5.54)		11.42 (5.32)		11.54 (5.43)

*Note. N* = 701

As a rule, each group was run by a minimum of two group leaders, and many also had back-up group leaders if one group leader was unable to attend all groups (*M* = 2.19, *SD* = 1.13). Descriptive information about the group leaders can be found in Table 2. Group leaders had various educational and professional backgrounds, with most working as health nurses. Notably, almost half had their primary workplace at school (40.2%). The experience in the field varied among the group leaders, and on average, the group leaders ran groups in the study for three waves.

**Table 2:** Sample characteristics for group leaders in the full sample, MFS and No-MFS conditions

	MFS		No-MFS		Full sample	
	*n* (%)	M (SD)	*n* (%)	M (SD)	*n* (%)	M (SD)
*Gender*						
Women	39 (95.1)		39 (92.9)		78 (94.0)	
Men	2 (4.9)		3 (7.1)		5 (6.0)	
*Profession*						
Health nurse	18 (43.9)		17 (40.5)		35 (42.2)	
Special education teacher	5 (12.2)		9 (21.4)		14 (16.9)	
Psychologist	6 (14.6)		3 (7.1)		9 (10.8)	
Teacher	3 (7.3)		1 (2.4)		4 (4.8)	
Child protection worker	1 (2.4)		1 (2.4)		2 (2.4)	
Other professions	8 (19.5)		11 (26.2)		19 (22.9)	
Clinical specialization ^a^	3 (7.3)		10 (23.8)		13 (15.7)	
Age		43.29 (10.30)		42.74 (9.85)		43.01 (10.02)
Years in the field		8.32 (7.14)		8.02 (6.69)		8.17 (6.88)
Number of courses		4.12 (2.50)		3.41 (2.61)		3.76 (2.57)
Number of groups in study		2.88 (1.27)		3.4 (1.31)		3.14 (1.31)

*Note.* MFS conditions *n* = 41, No-MFS conditions *n* = 42. ^a^Number/percentage that answered “yes”

### The MFS and the ROM Procedure

The MittEcho (own translation: MyEcho) MFS, was developed in collaboration with the University of Oslo, and meets the strictest data security requirements for health data in Norway when it comes to collecting, storing, and processing feedback data (Neumer et al., 2021). The system was developed for the present study and had not previously been tested with the user groups. There were therefore some technical problems that were addressed concurrently while conducting the study. Technical support was provided throughout the intervention period. The MittEcho system consists of two platforms, (1) the mobile application for collecting feedback data, the MittEcho app, and (2) a web-based desktop for displaying feedback results, the MittEcho publication portal. The MittEcho app was designed to be used by children and includes symbols such as emojis to complement the response categories, and a minimum of written texts. The app contained two types of measures, a brief symptom questionnaire and an idiographic measure where children could enter their own personal goals (up to three goals) and evaluate progress on these goals. The symptom measure was six questions of symptoms of anxiety and depression from the internalizing subscale of the Behavior and Feelings Scale for youth (BFS; Weisz et al., 2019). The MittEcho app did not display the responses for children to see themselves, however they would collect a star for each measure completed. The MittEcho publication portal required authentication of electronic proof of identity for group leaders to access data. The portal displayed BFS scores and self-evaluation of goal progress from the children in individual graphs over time. The publication portal also included a description of the measurements, instructions of how to interpret the graphs, and tips on ways to identify children whose symptoms were worsening or not improving.

Group leaders at the sites that were assigned to MFS conditions participated in a two-hour training on how to use the MittEcho system after receiving training in Emotion. The MFS training provided an introduction into how to use the app, the publication portal, and guidelines for how to interpret the feedback data and suggestions for how to implement it in the groups. Because of the group-format and that the primary intervention to a large degree dictated the session agendas, group leaders were instructed to monitor for NOT and prioritize such cases for follow-up. Group leaders were given additional information material: a short user manual, and instructional videos to learn on their own. They also had access to technical support from the research team, and MFS could be discussed in the regular supervision accompanying Emotion.

At the start of each new group, just prior to the first group session, parents of the participating children received an e-mail from the research team with instructions to download the MittEcho app to a mobile device that was available to the children. Group leaders also received an e-mail reminding them to implement the app in the group, help children log in to the app, and support them in making goals. Both parents and group leaders were instructed to help follow up children’s MFS use throughout the intervention period, though no data were collected on how this was done. The MittEcho app also sent notifications each week to remind children to answer questions and rate goal progress. Children used the app on their own smartphone, school tablet computer, or on a parent’s smartphone. The measures were to be answered between group sessions. Approximately three weeks into the intervention, the group leaders received another e-mail from the research team reminding them to monitor the children’s responses via the publication portal. The supervisors were also reminded by the research team to thematize the MFS in the bi-weekly supervision with the group leaders.

### Measures

The primary outcome was change in scores of depressive and anxious symptoms pre- to post intervention and was assessed using the *Mood and Feelings Questionnaire – Short version for children* (SMFQ; Angold et al., 1995), and the *Multidimensional Anxiety Scale for Children* (MASC; March et al., 1997). The SMFQ was designed to be a brief, but psychometrically valid tool for assessing depressive symptoms in children and adolescents. The scale consists of 13 self-report items rated on a three-point Likert-scale (0 = “*not true*”, 1 = “*sometimes*”, 2 = “*true*”). The SMFQ short scale has performed as well as the longer version in discriminating clinical samples, and it has good predictive and discriminant validity compared to other measures of depression (Angold et al., 1995). It has also showed good internal reliability (Cronbach’s α = 0.85). Internal reliability for outcome variables in the present study are shown in Table 3.

**Table 3:** Descriptive statistics for continuous outcome variables and predictors in the full sample, MFS conditions, and No-MFS conditions

	MFS		no-MFS		Full sample		50th Percentile	Range	Ω
	M	SD	M	SD	M	SD			
MASC change ^a^	12.60	18.06	10.10	17.19	11.40	17.68	11	[− 42, 82]	[0.62, 0.82]
SMFQ change ^b^	2.52	6.39	2.55	5.92	2.53	6.16	3	[− 18, 25]	0.85
User satisfaction Emotion ^c^	7.55	2.06	7.70	1.81	7.62	1.94	8	[1, 10]	0.87
Implementation Index	24.27	24.23	0	0	12.43	21.15	0	[0, 100]	
Child attendance Emotion	87.88	17.17	83.79	21.99	85.89	19.76	93.75	[0, 100]	
GL experience ^d^	0.18	0.95	−0.15	1.00	0.02	0.99	−0.04	[− 2.01, 3.06]	

*Note.* Statistics based on original sample values. *N* = 701 (n _MFS_ = 359, n _no−MFS_ = 342). MASC and SMFQ internal reliability are based on pre-intervention scores, while User satisfaction Emotion is based on post-intervention scores. Intervention format (blended): MFS = 44.3%, no-MFS = 49.7%. Parental involvement (high): MFS = 42.6%, no-MFS = 43.6%. MASC = Multidimensional Anxiety Scale for Children; SMFQ = Mood and Feelings Questionnaire – Short version for children; GL = group leader. ^a^*N* = 633. ^b^*N* = 632. ^c^*N* = 628. ^d^ GL experience on group level with child as unit

The MASC consists of 39 items that measures four dimensions of child anxiety: physical symptoms, social anxiety, harm avoidance, and separation anxiety (March et al., 1997). The items are rated on a four-point Likert scale (0 = “*never true about me*”, 1 = “*rarely true about me*”, 2 = “*sometimes true about me*”, 3 = “*often true about me*”). The factor structure has been shown to be invariant across age and sex, in a Norwegian sample (Martinsen et al., 2017). The scale has demonstrated predictive validity, and good test-retest reliability in a Norwegian sample (Villabø et al., 2012), and internal reliability of the factors ranged from 0.61 to 0.86 (Cronbach’s α; Martinsen et al., 2017).

The secondary outcomes were user satisfaction with Emotion, and intervention dropout. The children’s satisfaction was assessed at post-intervention using five questions adapted from the Stigma and Evaluation Sheet (Rapee et al., 2006). The five items were combined to form a mean user satisfaction score. The items were rated on a 10-point scale (1 = “*not good/not at all”*, 10 = “*very good/very much”*). Intervention dropout, also referred to as therapy dropout, was coded based on study logs of reasons for drop-out. For many cases it was possible to code dropout or missingness that was unrelated to the intervention, like illness at post-measurement or change of school during the intervention. The coding was dropout/no dropout, and 47 cases were classified as intervention dropouts.

Degree of MFS utilization was the primary predictor of interest and was measured using an adapted version of the Bickman et al. (2016) Implementation Index. In the present study the children’s response-rate (C-MFS dose) was calculated by the total number of weeks during the intervention they completed measures in the MittEcho app. This operationalization is similar to the previous studies, as children could respond a maximum of once per week. The group leader viewing-rate (GL-MFS dose) had to be adapted on several accounts due to the nature of the publication portal logs. First, GL-MFS dose could only be calculated on a group level as the portal gave access to all children’s data at once. Further, to calculate a ratio, login activity had to be compared to a maximum, and as group leaders could view all the children’s feedback in a single login session, the maximum dose was set to one login per week by either of the group leaders. Some group leaders logged in more than once a week, however, checking the feedback once a week was the minimum that was expected, and we considered routinely viewing the results throughout the intervention as more important for MFS dose and for discovering NOT’s. Additionally, the publication portal had some technical problems, and repeated logins could be reflective of this instead of a higher dose. In general, the intervention period lasted eight weeks, though this varied among the sites due to cancelled or delayed sessions. The data collection from the children was unable to be paused during these breaks, thus creating the possibility for higher maximum measurement points. Yet, dividing the maximum number of weeks accounted for variability in intervention length. The C-MFS dose and GL-MFS dose were then multiplied to form a composite measure, and further multiplied by 100 to create the Implementation Index ranging from 0 to 100, where 0 indicates no implementation and 100 equals maximum implementation. With this formula, both user-groups’ use of MFS becomes essential for the implementation of feedback. For example, in a scenario where the child completed all possible measures, but neither of the group leaders had reviewed the feedback, the resulting Implementation Index would be 0.

Demographic variables for children and their attendance-rate at Emotion were also included in analyses. Parents reported age and sex (“boy” or “girl”) of their child. Children’s attendance at group sessions was registered by group leaders at each session; the attendance-rate was calculated by dividing attendance by the total number of sessions.

Group leader experience was measured by averaging experience and qualifications among the active group leaders. Experience was assumed to be a formative construct and was measured as a weighted composite variable. A naïve Principal Component Analysis with extraction of one factor explained 35% of the variance. The components and associated loadings were: (1) number of years working in the field (λ = 0.59), (2) number of courses in mental health related topics and interventions, such as CBT or other mental health interventions, (λ = 0.67), (3) a cumulative count of the number of times they led groups within the Echo-study (λ = −0.28), and (4) clinical specialization (λ = 0.71). Correlations among the variables were small (*r* between − 0.14 and 0.21), and each variable was transformed to z-scores prior to calculation of the composite variable.

### Statistical Methods

The statistical analyses were conducted using IBM SPSS Statistics Version 27 (IBM Corporation, 2022). The primary hypotheses were assessed using a multilevel linear mixed modeling approach, which accommodates the hierarchical structure of the data, wherein children were nested within therapy groups. We first assessed a random intercept model to examine the presence of treatment effect dependencies within therapy groups. Then a random slope model was studied. This model informed us about whether MFS dose-response effects varied between therapy groups. Further interaction effects between the Implementation Index and covariates were investigated to reliably interpret main effects. Interactions among Implementation Index and children’s age, sex, baseline symptoms, Emotion attendance-rate, group leader experience, the two experimental conditions, delivery format, and parent involvement were tested collectively. Model comparison was done when assessing parameters on the same level using χ^2^ difference test between the −2 log likelihood values of the models, with degrees of freedom corresponding to the disparity in the number of parameters between them. The better fitting model was chosen for interpretation and presentation. The first choice for the analyses was Restricted Maximum Likelihood Estimation (REML). However, deviance testing was performed on Maximum Likelihood (ML) estimated models as it allows for testing of the fixed effects, as opposed to REML where only variance components can be tested (Singer & Willett, 2003). For model goodness-of-fit, both conditional and marginal pseudo R^2^ was reported to get an impression of the contribution of fixed and random effects and the fixed effects only (Nakagawa & Schielzeth, 2013).

For the secondary hypothesis of MFSs’ effect on intervention dropout, the Implementation Index could not be used due to risk of confounding, and in the analysis MFS-condition was chosen as predictor instead. Because of the binary outcome variable of dropout vs. no dropout, a logistic regression was chosen. With a suspicion of group dependency for dropout, supported by naïve intraclass correlation coefficients (ICC) estimate, group was modeled with a generalized linear mixed model.

When encountering estimation problems, the maximum iterations were increased, or ML estimation was tested. If no convergence was achieved, the random effects part of the model was simplified.

Post-intervention, there were missing MASC (9.7%), SMFQ (9.8%), and user satisfaction (10.4%) data. For the MASC and SMFQ, both multiple imputation (MI) and simulation were used to impute the missing values to mitigate bias in estimation. Because of a lack of good predictors for user satisfaction, the missing values could not be imputed, and listwise deletion was used in the analysis of the main outcome. The reasons for dropout and missing replies were documented throughout the study and cases were classified according to missing at random or completely at random (MAR/MCAR) and not missing at random (NMAR; Rubin, 1976 as cited in McKnight et al., 2007). Out of the cases with missing MASC and SMFQ data, 20 cases were classified as MAR or MCAR and for these we used multiple imputation (25 datasets) with predictive mean matching using the five closest cases. As ICC for MASC and SMFQ indicated that dependency within groups should be considered, the multiple imputation model used fixed effects imputation and included group as a level. Relevant demographic, individual, and group characteristic variables were included as predictors. For the 47 cases classified as NMAR, the post intervention scores were simulated using a normal distribution with an expected effect of 0. This procedure was repeated 25 times to mimic the multiple imputation procedure.

To ensure high external validity of the results, all cases were included in analysis whenever possible. This meant that participants who did not use the MFS were given a value of zero on the Implementation Index. This was true for both those in the MFS conditions and those in no-MFS conditions.

## Results

### Descriptives

Descriptive statistics for continuous outcome variables of primary and secondary hypotheses are displayed in Table 3. As anticipated when using simulation with an expected change of 0 for the NMAR cases, the pooled means for both post-scores on MASC and SMFQ were slightly higher than in the original dataset (MASC post: *M*_original_ = 58.30, *M*_pooled_ = 58.90, SMFQ post: *M*_original_ = 9.00, *M*_pooled_ = 9.20), resulting in slightly more conservative estimates for the change scores in anxiety and depression in the analysis. Girls had significantly higher scores on anxiety pre-intervention than boys (*t*(551.2) = 5.13, *p* < .001), and on depression scores (*t*(586.7) = 2.24, *p* = .026). A total of 47 cases were classified as possible intervention dropout, with 6.2% in the MFS groups and 8.2% in the non-MFS groups. In the total sample there were no dropouts in 88 of the child groups, one dropout in 30 groups, two dropouts in four groups, and four dropouts in two groups.

**Fig. 1:**
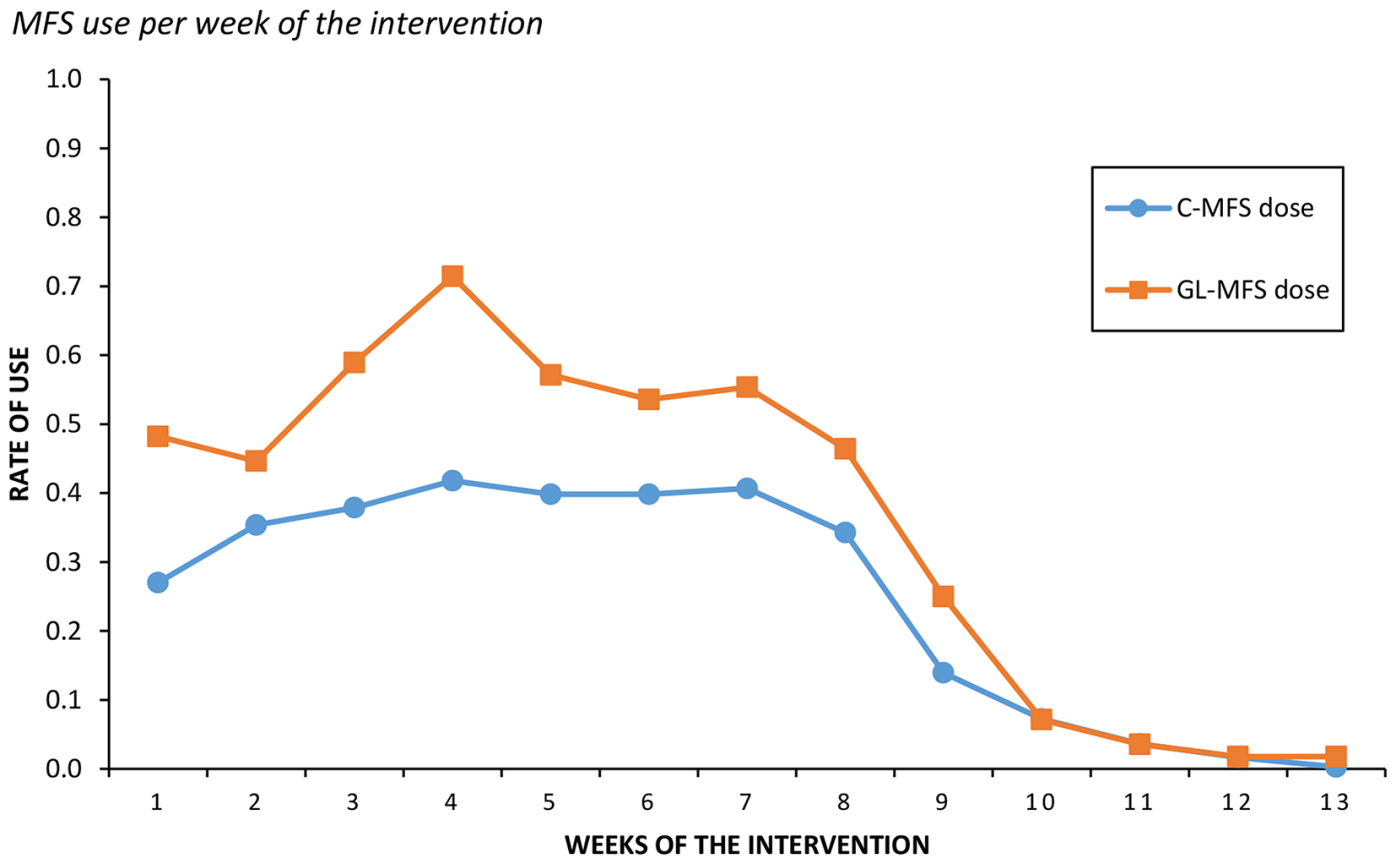
MFS use per week of the intervention *Note*. This figure displays the average child respondent-rate (C-MFS dose) and group leader viewing-rate (GL-MFS dose), each week of the intervention period. In general, the Emotion intervention spanned across eight weeks, but a few groups lasted longer due to delays and the MFS app would not be paused during these delays. C-MFS dose is calculated per individual (*N* = 359), while GL-MFS dose is calculated on a group level (*N* = 56)

The mean score on the Implementation Index in the MFS groups was 24.27 (*SD* = 24.23), out of 100. Among the children in the MFS condition, 78.8% answered at least one measure, 71.9% answered at least two measures, while 25.3% answered at least eight measures. The mean use among the children was 0.40 (*SD* = 0.31), indicating that on average the children answered 40% of the possible measures. Figure 1 displays the mean C-MFS dose and GL-MFS dose throughout the weeks of the intervention. Group leaders logged into the publication portal a total of 462 times during the intervention period, where approximately 7.5% were duplicate logins by the same person less than 10 min apart. In 25 of the groups one group leader accessed results at least once, and in 23 groups two group leaders accessed the results at least once, yet it seemed that one of the group leaders was the primary user. In four of the MFS-groups (22 children), neither of the group-leaders logged into the children’s feedback in any of the intervention weeks. For the group leaders in the MFS condition the mean use was 0.52 (*SD* = 0.30), meaning that on average at least one of the group leaders logged into the results portal 52% of the possible weeks.

### Results of the MFS Implementation Index on Anxiety Outcome

An initial model including only a random effect of group intercept indicated that a significant portion of the total variation in the MASC total change score (ICC = 0.077, *p* = .020) was attributed to between-group differences, suggesting the potential benefit of incorporating therapy group-level predictors. Next, a model including a random slope of the Implementation Index was assessed, and a difference test using −2 log-likelihood (−2LL) of a full model including fixed effect and random slope for the Implementation Index (−2LL = 5422.42) and a reduced model including only the fixed effect (−2LL = 5423.22) indicated that including the random slope did not improve fit of the model (χ^2^(1) = 0.80, *p* = .371) and was therefore not included in further analyses.

Six variables were assumed to moderate the dose-response effect: child symptom level of anxiety at screening, attendance-rate at Emotion, delivery format of Emotion, parental involvement, sex of child, and group leader experience. Consequently, six two-way interactions involving the Implementation Index were included in the full model. The full model (−2LL = 5266.26) was tested against a restricted model containing only the main effects (−2LL = 5269.49). The difference was non-significant, indicating that the interaction terms did not contribute to better model fit (χ^2^(6) = 3.23, *p* = .779).

Results from the final mixed model for anxiety outcome are shown in Table 4. The MFS Implementation Index did not significantly predict symptom change in anxiety from pre to post intervention (*p* = .295). In this model, pre-intervention anxiety scores, attendance-rate at Emotion, and sex of child significantly predicted changes in MASC scores post intervention. Having higher anxiety scores on MASC before the intervention (*b* = 0.52, *t*(624.00) = 12.08, *p* < .001), and having higher attendance-rate at Emotion (*b* = 0.09, *t*(372.17) = 2.45, *p* = .014) were associated with positive and larger changes in anxiety scores post intervention, and being a boy was associated with smaller changes in anxiety scores (*b* = − 3.98, *t*(623.70) = − 3.07, *p* = .002).

**Table 4:** Main effects of the Implementation Index and covariates on change in anxiety symptoms from pre to post intervention

Effect	Estimate	SE	95% CI		*p*
			*LL*	*UL*	
*Fixed effects*					
Intercept	−30.82	11.35	−53.07	−8.58	0.007
Implementation Index	0.03	0.03	−0.03	0.10	0.295
MASC pre intervention	0.52	0.04	0.43	0.60	< 0.001
Sex of child ^a^	−3.98	1.30	−6.52	−1.43	0.002
Child age	−0.01	0.97	−1.92	1.90	0.991
Attendance Emotion	0.09	0.04	0.02	0.15	0.014
Delivery format Emotion^ab^	−0.54	1.44	−3.36	2.28	0.707
Parental involvement^ab^	0.92	1.47	−1.96	3.80	0.523
GL experience ^b^	0.48	0.74	−0.98	1.93	0.295
*Random effects*					
Within-study variance	230.30	13.93	202.99	257.61	< 0.001
Between-study variance	16.22	7.76	1.02	31.43	0.036

*Note.* Pseudo R2: conditional = 0.27, marginal = 0.21. CI = confidence interval; LL = lower limit; UL = upper limit; MASC = Multidimensional Anxiety Scale for Children; GL = group leader. ^a^Reference category is the lowest value: Sex of child: girls = 0, boys = 1; Delivery format: blended = 1, group = 2; Parental involvement: low = 1, high = 2. ^b^Variables measured on group level

### Results of the MFS Implementation Index on Depression Outcome

The first model including only a random effect of group indicated that intercepts did not vary significantly (ICC = 0.029, *p* = .391). However, it was decided to keep the group level in the analysis for more precise estimation of the fixed effects. Next, modeling a random slope for the Implementation Index, a difference test of a full model including fixed effect and random slope for the Implementation Index (−2LL = 4089.52) and a reduced model including only the fixed effect (−2LL = 4089.72) indicated that model fit was not significantly improved (χ^2^(1) = 0.20, *p* = .654), and random slope was not included in further analyses.

As with the MASC outcome, moderators of the association between the Implementation Index and symptom reduction on depression scores were assessed before investigating main effects. The full model tested six two-way interactions with the Implementation Index involving child symptom level of depression at screening, attendance-rate at Emotion, delivery format of Emotion, parental involvement, sex of child, and group leader experience. Comparing the full (−2LL = 3921.96) and restricted (−2LL = 3928.70) model revealed no significant difference (χ^2^(6) = 6.75, *p* = .345), indicating no better model fit when including the interaction terms.

Results from the final mixed model for depression symptoms as outcome are shown in Table 5. The MFS Implementation Index did not significantly predict changes in depressive symptoms from pre to post intervention (*p* = .877). In the final model, only the pre-intervention depression levels and the attendance-rate at Emotion significantly predicted changes in depression scores. Higher depression scores at screening were associated with positive, and greater, changes in depression scores post-intervention (*b* = 0.51, *t*(615.45) = 12.94, *p* < .001). The analysis also indicated that having a higher attendance-rate was associated with a positive change in depression (*b* = 0.04, *t*(360.61) = 3.10, *p* = .002).

**Table 5:** Main effects of Implementation Index and covariates for change in depression symptoms from pre to post intervention

Effect	Estimate	SE	95% CI		*p*
			LL	UL	
*Fixed effects*					
Intercept	−4.43	3.78	−11.83	2.98	0.241
Implementation Index	2*10^− 3^	0.01	−0.02	0.02	0.877
SMFQ pre intervention	0.51	0.04	0.43	0.58	< 0.001
Sex of child ^a^	−0.40	0.44	−1.26	0.46	0.357
Child age	−0.19	0.34	−0.86	0.47	0.569
Attendance Emotion	0.04	0.01	0.01	0.06	0.002
Delivery format Emotion^ab^	−0.50	0.50	−1.49	0.48	0.241
Parental involvement^ab^	0.07	0.52	−0.94	1.09	0.886
GL experience ^b^	0.01	0.26	−0.51	0.52	0.972
*Random effects*					
Within-study variance	27.55	1.70	24.21	30.89	< 0.001
Between-study variance	2.14	1.02	0.14	4.14	0.036

*Note.* Pseudo R2: conditional = 0.29, marginal = 0.22. CI = confidence interval; LL = lower limit; UL = upper limit; SMFQ = Mood and Feelings Questionnaire – Short version for children; GL = group leader. ^a^Reference category is the lowest value: Sex of child: girls = 0, boys = 1; Delivery format: blended = 1, group = 2; Parental involvement: low = 1, high = 2. ^b^Variables measured on group level

### Results of the MFS Implementation Index on User Satisfaction

An initial model including only random effect of group showed significant intercept variance for the therapy groups (ICC = 0.128, *p* < .001). Adding a random slope effect led to estimation problems of the model, and changing estimation method and increasing maximum iterations did not amend the problem, and this random effect was not included in further analyses.

Further, a full model of eight two-way interactions with the Implementation Index was tested. The moderators tested were the following variables: attendance-rate of Emotion, delivery format of the Emotion, parental involvement, age and sex of child, group leader experience, and the change scores of both MASC and SMFQ. The test of the full (−2LL = 2566.14) and the restricted (−2LL = 2572.33) model revealed no significant difference (χ^2^(8) = 6.19, *p* = .626), indicating that the interactions did not improve model fit.

The final model for user satisfaction of Emotion is shown in Table 6, and as can be seen the MFS Implementation Index did not predict the children’s satisfaction with the Emotion program significantly (*p* = .863). Children’s satisfaction with Emotion post intervention was significantly associated with children’s age and the change score in depression. The association with children’s age indicated that the younger children rated satisfaction with Emotion higher than the older children (*b* = − 0.32, *t*(367.18) = − 2.61, *p* = .009). Further, children experiencing a larger change in depression scores was more satisfied than those experiencing less change (*b* = 0.04, *t*(606.50) = 2.46, *p* = .014).

**Table 6:** Main Effects of Implementation Index and covariates on user satisfaction with the Emotion

Effect	Estimate	SE	95% CI		*p*
			*LL*	*UL*	
*Fixed effects*					
Intercept	10.72	1.46	7.87	13.54	< 0.001
Implementation Index	−1*10^− 3^	4*10^− 3^	−0.01	0.01	0.863
MASC change score	−0*10^− 3^	0.01	−0.01	0.01	0.949
SMFQ change score	0.04	0.01	0.01	0.06	0.014
Sex of child ^a^	0.03	0.16	−0.28	0.34	0.849
Child age	−0.32	0.12	−0.57	−0.08	0.009
Attendance Emotion	−4*10^− 3^	0.01	−0.01	0.02	0.681
Delivery format Emotion^ab^	−0.25	0.19	−0.62	0.13	0.193
Parental involvement^ab^	−0.07	0.20	−0.46	0.31	0.711
GL experience ^b^	−0.10	0.10	−0.30	0.09	0.304
*Random effects*					
Within-study variance	3.25	0.20	2.85	3.65	< 0.001
Between-study variance	0.41	0.14	0.14	0.68	0.003

*Note.* Pseudo R2: conditional = 0.27, marginal = 0.21. CI = confidence interval; LL = lower limit; UL = upper limit; MASC = Multidimensional Anxiety Scale for Children; SMFQ = Mood and Feelings Questionnaire – Short version for children; GL = group leader. ^a^Reference category is the lowest value: Sex of child: girls = 0, boys = 1; Delivery format: blended = 1, group = 2; Parental involvement: low = 1, high = 2. ^b^Variables measured on group level

### Results of the MFS Implementation Index on Intervention Dropout

A naïve estimation indicated some dependency within child groups (ICC = 0.064) and a generalized linear mixed model including group as a second level was attempted. Yet, there were problems estimating this model which was likely due to the low number of positive events and the model was simplified by removing the random intercept for group. A model including baseline symptoms of anxiety and depression as covariates was also tested, however, a stepwise logistic regression indicated that including the covariates did not improve fit of the model (χ^2^(2) = 4.51, *p* = .105). Finally, a logistic regression including only MFS/no-MFS condition as predictor of intervention dropout revealed no significant difference between the MFS and no-MFS groups (*B* = 0.281, *SE* = 0.304, *p* = .355, *OR* = 1.32, 95% CI = [0.730, 2.401]).

## Discussion

The aim of the present study was to investigate if the implementation of ROM using a novel MFS moderated outcomes for children participating in an indicated group-based intervention. The main hypotheses was that a higher degree of MFS implementation, as measured by higher scores on the Implementation Index, would be associated with greater reductions in symptoms of anxiety and depression post intervention. The secondary hypotheses were that higher implementation of MFS should be associated with greater child satisfaction with the primary intervention; Emotion, and that there should be less dropout during the intervention among the children using MFS. There was no support in the present study for either the main hypotheses nor the secondary hypotheses.

The analyses did reveal that severity of both anxiety and depression symptoms at screening significantly predicted a reduction in symptoms post intervention. In essence, the children with the highest symptoms responded best after participating in Emotion. Though, it must be said that this effect can partly be seen because high scorers have a higher potential for change than low scorers. Further, higher attendance at Emotion was associated with a larger change in both anxiety and depression. This is a promising finding for the effects of Emotion that such a dose-response effect exists, however, as there was no control group, the support for the effect of the intervention should be interpreted with caution. Further, the sex of the child was significantly associated with change in anxiety symptoms, with girls showing larger changes in anxiety scores. This could be connected to responsiveness to the intervention; however, girls typically score higher on anxiety, as was true in the present study, and thus have larger potential for symptom reduction (Racine et al., 2021).

For the secondary hypotheses, children’s satisfaction with the Emotion program, age, and change-scores in depression, but not anxiety, were associated with higher satisfaction. It is perhaps not surprising that individuals who reported that the intervention helped with their symptoms also rated satisfaction with the intervention higher; but it was surprising that this effect was only seen for depression and not anxiety. Younger children rated satisfaction with the intervention higher, and it is not clear why. No variables included in the analysis predicted dropout; this was not surprising, as the study was not powered to find effects when there were so few positive events.

The results from the current study were not in line with others who have investigated dose-effects of ROM and MFS in youth using the Implementation Index (Bickman et al., 2016; Sale et al., 2021). One reason might be the calculation of the Implementation Index which differed from Bickman et al. (2016) and Sale et al. (2021), particularly the group leader viewing-rate. In these previous studies, the response from each child or caregiver would generate a report and they could sum up the rate of reports viewed by therapists. In the present study, only activity logs from the publication portal were available. Thus, viewing of results from each individual child could not be differentiated, and it was necessary to make some adaptations to get a group leader viewing rate. This might have resulted in an imprecise estimation of group leader dose. However, our adaptation meant that we could retain the Implementation Index’s range from 0 to 100 as in the previously mentioned studies. Secondly, the mean Implementation Index was lower (*M* = 24.27, *SD* = 24.23) in the current study than the previous studies (Bickman et al., 2016: clinic *R* = 27%, clinic U = 34%; Sale et al., 2021: caregiver report = 48.09%, youth report = 50.66%), and there was large variation in use. The level of use and the skewed distribution of the Implementation Index in our study did, however, resemble the sample from Bickman et al. (2016) in which no significant effect of MFS was found. This could point to a minimum threshold of implementation for ROM to have effects on symptom reduction. Differences in inclusion criteria may also explain the lower level of implementation in the present study. Both Bickman et al. (2016) and Sale et al. (2021) included only children that had at least one MFS report in analyses, while in the present Implementation Index estimate all cases randomized to the MFS-conditions were included. Furthermore, Sale et al. (2021) and Bickman et al. (2016) modeled rate of change using the MFS progress measure as outcome, whereas in the present study, independent measures were used to assess the difference pre to post intervention. Additionally, the length of intervention (i.e., number of sessions), age of children, the MFS and progress measure, and the treatment format were not the same between the studies.

The age of the reporter group in the present sample was younger than what has been typically included in studies with ROM, and there were no other reporters on child progress, such as caregivers or therapists (Tam & Ronan, 2017). Yet, in other studies with multiple reports on MFS or outcome measure, children and other reporters disagree (Meyer et al., 2001). For instance, parents seem to underreport children’s internalizing symptoms compared to self-reports and intervention effects are not always seen across informants (Martinsen et al., 2019). Furthermore, the measure used to monitor children in the present study has been validated for the present age group (Weisz et al., 2019), which together with the age-appropriate rating (e.g., smiley faces in the app), indicates that children are able to assess their symptoms. Idiographic measures also seem like a psychometrically sound approach for this age group (Weisz et al., 2011).

A general limitation with conceptualizing ROM and MFS use with the Implementation Index is that it does not include whether the feedback was used in decision-making or to make adaptations to the intervention (Bickman et al., 2016). While measuring child response-rate and group leader viewing-rate gives some indication to what degree the MFS was used, it does not necessarily reflect whether the results were addressed in any way. Considering the proposed core components of ROM practice, we lacked data on the final, and most important component according to research: incorporating the feedback into therapy (Barkham et al., 2023; Krägeloh et al., 2015). The ROM literature is increasingly focusing on what therapists do with the information (Brooks Holliday et al., 2020; Casline et al., 2022; Kwan et al., 2021; Laver et al., 2024). Therapists actively discussing feedback in the session or reporting using feedback to plan or adjust treatment strategies seems less common despite being connected to perceptions of ROM utility for both the therapist and client (Brooks Holliday et al., 2020; Casline et al., 2022; Kwan et al., 2021). In the Echo study, many group leaders indicated that they were unsuccessful using feedback in the sessions (Haug et al., 2024). On that note, it is also important that they be given freedom to make changes (Davidsen et al., 2017). Strict manuals or treatment protocols that do not allow for flexibility makes it challenging to exploit the potential of ROM. The intervention in the present study also used a manual-based approach, that together with the frames of the research study, may have limited the flexibility to utilize the feedback.

The current results were in line with findings from Shechtman and Sarig (2016), who examined feedback in group-based mental health interventions for children in a school setting. Their RCT of 220 children (age 10 to 18 years) in feedback or no-feedback condition, showed no effect on internalizing or externalizing symptoms. They neither found a difference in the change rate over time, nor an effect on pre-post difference scores between feedback and no-feedback conditions. Yet, they did not include any measure of dose or fidelity of the feedback intervention and a lack of implementation may have explained their results. The authors attributed the intervention´s group-format as the reason for not finding comparable results to studies of individual treatment of children.

The group-based format may indeed present additional challenges, especially with children. Focusing on the needs of several individuals at once while delivering program content may strain the group leaders’ attention and thus limit their capacity and time to attend to feedback from the group. Others argue, however, that it is precisely the intensiveness of group meetings that warrants the use of MFS, because this setting makes it difficult to monitor the progress of each participant without the use of ROM (Koementas-de Vos et al., 2018). Not on track cases also seems to be more common in group settings, which also suggests the utility of ROM in groups (Alldredge et al., 2020). On one hand it seems reasonable to use ROM in groups to identify the children that need additional support, but on the other hand, there are practical barriers for group leaders to relate to the feedback of several children at once. Future research should address these barriers to see if there is a way to provide meaningful and effective feedback in an efficient and practical manner in these specific settings.

The randomized factorial design with MFS/no-MFS conditions, and a large national sample with high ecological validity were strengths in the current study. In addition, this study investigated the degree of implementation of MFS using the Implementation Index which was developed and used in related research and considers the fidelity of the intervention and thus increased the validity of the findings (Bickman et al., 2016). Further, we examined the degree of implementation of MFS on outcome measures independent from the MFS measures. The large sample also provided sufficient power to have confidence in the results.

There were also some methodological limitations to this study that may explain why we did not replicate positive results found elsewhere. First, in our operationalization of the Implementation Index, we did not utilize all data from the publication portal logs. Further, we could not separate views on individuals’ feedback from the logs. This may have reduced variance in group leader use and power to detect effects. In part, to adapt the data to fit the calculations of the Implementation Index, our decision was driven by a need to acknowledge the instructions given to the group leaders at the time of training, and to reflect that the intervention introduced new content each week. It was therefore our view that it was more important that the group leaders checked feedback periodically over the course of the intervention, rather than at concentrated intervals. However, it is unclear if a different operationalization of dose, like a frequency measure of group leader use instead of viewing-rate, could have affected the results. Secondly, we lacked quantitative data on what group leaders did with the feedback, which seems to be the most crucial part of ROM practice (Krägeloh et al., 2015).

While the study was adequately powered, the effect estimates for MFS (Cohens *d =* 0.25, *α* = 0.80, *p* = .05; Neumer et al., 2021) may have been optimistic compared to estimates found in recent metanalyses. Though higher effects are reported for children and young people it is based on very few studies and effect sizes of 0.11–0.15 are to be expected (de Jong et al., 2021; Rognstad et al., 2023). Further, power analysis assumes that the experimental conditions are implemented as intended, yet as the present article has shown, it was not, meaning that there would be less power to detect differences. Looking for a smaller effect might have changed the results of the primary hypothesis in this study; however, it is worth a discussion at what magnitude and in which setting ROM and MFS has a practical effect; and when its utility outweigh the costs and effort of developing and implementing it.

Another methodological limitation was the tentative group leader experience construct used in the present study. Therapist’s experience is inconsistently related to outcome of ROM and use of it, and important to include in analysis (de Jong et al., 2012; Rye et al., 2019). While there are benefits of composite variables, combining variables with low associations among them may increase variance and power (Brown, 2015; Kline, 2016). The variables available in the present study do not comprise all facets of experience, and the this composite variable is likely an incomplete representation of group leader experience. The relative contribution of the indicators should also be weighed based on other sources of information than a single PCA.

There were also limitations to the present MFS and its implementation. The fact that a novel MFS was used in this study could have contributed to not finding a statistically significant effect. Recent evidence suggests that the type of system, certain functions, and instruments moderate effect sizes, and more established systems and measures show larger effects (de Jong et al., 2021; Rognstad et al., 2023). The MittEcho MFS was tested for the very first time in the present study, and it had not been piloted prior to the study. This had some consequences for the stability of the system and its usability. For example, there were technical problems with both the MittEcho app and MittEcho publication portal early in the study, and there were several fixes and updates to the technical solution during the study period. Further, the publication portal did not include alerts or case spesific decicional support to help the group leaders identify NOT cases and chose a course of action. Meta-analysis indicated that such functions are more effective than presenting therapists with raw data (de Jong et al., 2021). Alerts and decision-making tools may be especially helpful as studies find that most of the variation in ROM use can be attributed to therapist behavior (Bickman et al., 2016; Sale et al., 2021). On the other hand, acceptability, and availability for the children in the present study seemed good. Most of the children had access to smartphones and logged into the app and replied at least once.

The low implementation rate in this study could be connected to the amount of training and supervision in ROM and MFS that was provided. The training in ROM was brief (two hour workshop), and the group leaders recived training in Emotion and other study conditions simultainously. It might have been much to learn at once, especially as ROM procedure was not integrated in the Emotion program manual. Supervision was provided yet occurred in a group-format and did not focus solely on ROM. While more training may be desirable, brief trainings are perhaps the best one can expect within the school setting, as essential personnel are taken away from their usual tasks during more comprehensive training. Despite the brief training the group leaders had access to user manuals, online training videos, and technical support and supervision. In an ideal world, a more targeted training on ROM and MFS that included practice, rehearsal, and supervision dedicated only to ROM could have increased the implementation rates in our study. Another barrier for implementation in the municipal service setting could be that for many group leaders there are limited opportunities for collaboration and discussing feedback. Small units, being employed in different sections, and strict timetables with competing tasks are some of the challenges. Providing spaces for collaboration and supervision may be a way to overcome this.

Based on the results of the current study, we cannot claim that using the MittEcho system has an effect on symptom reduction, user satisfaction, or treatment dropout for children attending an indicated and group-based intervention. This result was robust even when the degree of implementation and the use of the MFS was accounted for. However, there might be other reasons for including routine feedback into services than for immediate effectiveness on symptoms. For example, administrative uses or policy reasons (Oanes 2017; Rousmaniere et al., 2020). ROM could support young people’s involvement in treatment and decision-making on matters that concern them, or in the development and design of mental health services for youth.

Though we want to be cautious recommending ROM using MFS based on it having effects on reducing child mental health symptoms in indicated group settings, we can offer some lessons learned on implementation: (1) The training should be comprehensive enough to reach a satisfying level of implementation but balance the time that personnel are away from other tasks. While facilitating implementation of ROM might lead to more use, research also indicates limited benefits of adding consultation beyond a certain amount (Lyon et al., 2022; Wray et al., 2023). (2) It is challenging to learn a new intervention and ROM, when ROM is not well integrated in the primary intervention. To reduce strain on group leaders and mitigate problems regarding what elements to prioritize, it is important that program developers incorporate ROM in the intervention program (Skeat & Perry, 2008). (3) A working environment that provides opportunity to reflect and discuss the feedback and ways of tailoring the intervention seems beneficial (Esmiol-Wilson et al., 2017). Routine supervision dedicated to feedback could help accomplish this. (4) The group setting may present some other challenges for using and incorporating ROM than in individual therapy (Gold & Kivlighan, 2018). Adapting MFS and ROM protocols specifically for the group setting might enhance its use and effectiveness in the group-format. (5) Developing MFS with focus on usability for group leaders, such as incorporating alerts and interpretive-, and decision-making support, may facilitate use of the feedback (de Jong et al., 2021) .

## Electronic Supplementary Material

Below is the link to the electronic supplementary material.

Supplementary Material 1

Supplementary Material 2

Supplementary Material 3

Supplementary Material 4

Supplementary Material 5
