# Supplementary material for: Dose-Response Effects of MittEcho, a Measurement Feedback System, in an Indicated Mental Health Intervention for Children in Municipal and School Services in Norway
Source: Adm Policy Ment Health. 2024 May 29;52(1):223–40. doi: 10.1007/s10488-024-01389-9 (PMC11703986; doi:10.1007/s10488-024-01389-9)
Supplement: Supplementary file 1 — Supplementary Material 1 [file 10488_2024_1389_MOESM1_ESM.docx]

**Supplementary 2**

**Table 8**

*Mean and Standard Deviation for Anxiety, Depression and User Satisfaction for the Full Sample, MFS Condition and No-MFS Condition*

|  | MFS | | No-MFS | | Full sample | | | |
| --- | --- | --- | --- | --- | --- | --- | --- | --- |
|  | *M* | *SD* | *M* | *SD* | *M* | *SD* | Scale Range | Omega |
| MASC change pre-post | 12.60 | 18.06 | 10.10 | 17.19 | 11.39 | 17.68 | [0, 117] | [.62, .82] |
| SMFQ change pre-post | 2.52 | 6.38 | 2.55 | 5.92 | 2.53 | 6.16 | [0­­, 26] | .85 |
| User Satisfaction Emotion | 7.55 | 2.06 | 7.70 | 1.80 | 7.62 | 1.94 | [0, 10] | .87 |

*Note.* MASC and SMFQ Omega value based on pre-intervention scores, while User satisfaction Emotion based on post-intervention scores. Range displays the range for the scale. MASC = Multidimensional Anxiety Scale for Children; SMFQ = Mood and Feelings Questionnaire – Short version for children.
