# Supplementary material for: Dose-Response Effects of MittEcho, a Measurement Feedback System, in an Indicated Mental Health Intervention for Children in Municipal and School Services in Norway
Source: Adm Policy Ment Health. 2024 May 29;52(1):223–40. doi: 10.1007/s10488-024-01389-9 (PMC11703986; doi:10.1007/s10488-024-01389-9)
Supplement: Supplementary file 2 — Supplementary Material 2 [file 10488_2024_1389_MOESM2_ESM.docx]

**Supplementary 1**

**Table 7**

*Descriptive Statistics and Correlations for Study Variables*

| Variable | *M* | *SD* | 1 | 2 | 3 | 4 | 5 | 6 | 7 | 8 | 9 | 10 | 11 |
| --- | --- | --- | --- | --- | --- | --- | --- | --- | --- | --- | --- | --- | --- |
| 1. MASC post | 58.30 | 17.22 | - |  |  |  |  |  |  |  |  |  |  |
| 2. SMFQ post | 9.00 | 6.06 | .54** | - |  |  |  |  |  |  |  |  |  |
| 3. User satisfaction Emotion | 7.62 | 1.94 | −.02 | −.16** | - |  |  |  |  |  |  |  |  |
| 4. Implementation Index | 12.43 | 21.15 | −.06 | −.03 | −.01 | - |  |  |  |  |  |  |  |
| 5. MASC pre | 69.56 | 14.81 | .44** | .19** | .04 | −.02 | - |  |  |  |  |  |  |
| 6. SMFQ pre | 11.54 | 5.43 | .23** | .47** | −.04 | −.02 | .44** | - |  |  |  |  |  |
| 7. Attendance Emotion | 85.89 | 19.76 | −.07 | −.13** | .04 | .19** | .04 | −.05 | - |  |  |  |  |
| 8. GL experience ᵃ | 0.02 | 0.99 | −.02 | .01 | −.08 | −.02 | .01 | .03 | −.05 | - |  |  |  |
| 9. Child age | 10.58 | 0.69 | −.02 | .03 | −.14** | .06 | −.06 | .01 | −.03 | −.19*** | - |  |  |
| 10. Child sex | 0.40 | 0.49 | −.18** | −.06 | .00 | −.08* | −.12** | −.09* | −.13** | .01 | −.06 | - |  |
| 11. Format Emotion ᵃ | 1.53 | 0.50 | −.08* | −.05 | .07 | .10** | −.13** | −.04 | .03 | .06 | −.03 | .03 | - |
| 12. Parental involvement ᵃ | 1.43 | 0.50 | .04 | .01 | .03 | −.02 | .02 | −.01 | −.06 | −.23*** | −.02 | .06 | .14** |

*Note.* Mean and SD for original values. Correlations with pooled estimates. MASC post: M pooled = 58.90; SMFQ post: M pooled = 9.20. N = 701 (MASC post, N = 633, SMFQ post, N = 632, User satisfaction Emotion, N = 628). MASC = Multidimensional Anxiety Scale for Children; SMFQ = Mood and Feelings Questionnaire – Short version for children; GL= group leader.

ᵃ Variables on group level.

* p < .05. ** p < .01. *** p < .001.
