# Supplementary material for: Dose-Response Effects of MittEcho, a Measurement Feedback System, in an Indicated Mental Health Intervention for Children in Municipal and School Services in Norway
Source: Adm Policy Ment Health. 2024 May 29;52(1):223–40. doi: 10.1007/s10488-024-01389-9 (PMC11703986; doi:10.1007/s10488-024-01389-9)
Supplement: Supplementary file 3 — Supplementary Material 3 [file 10488_2024_1389_MOESM3_ESM.docx]

**Supplementary 3**

**Table 9**

*Fixed, Random Effects and Model Fit Indices for Model A, B and Final Model (C) for the Dose-Response Effect of MFS Implementation on Changes in Anxiety Scores*

| Effects | Model A | | | Model B | | | Model C | | |
| --- | --- | --- | --- | --- | --- | --- | --- | --- | --- |
|  | estimate | *SE* | *p* | estimate | *SE* | *p* | estimate | *SE* | *p* |
| Fixed effects |  |  |  |  |  |  |  |  |  |
| Intercept | 10.62 | 0.78 | <.001 | 10.18 | 0.87 | <.001 | −30.82 | 11.35 | .007 |
| Implementation Index |  |  |  | 0.04 | 0.04 | .331 | 0.03 | 0.03 | .295 |
| MASC pre intervention |  |  |  |  |  |  | 0.52 | 0.04 | <.001 |
| Sex of child ^a^ |  |  |  |  |  |  | −3.98 | 1.30 | .002 |
| Child age |  |  |  |  |  |  | −0.01 | 0.97 | .991 |
| Attendance Emotion |  |  |  |  |  |  | 0.09 | 0.04 | .014 |
| Delivery format Emotion ^a^ |  |  |  |  |  |  | −0.54 | 1.44 | .707 |
| Parental involvement ^a^ |  |  |  |  |  |  | 0.92 | 1.47 | .532 |
| GL experience |  |  |  |  |  |  | 0.48 | 0.74 | .521 |
| Random effects |  |  |  |  |  |  |  |  |  |
| Residual | 282.30 | 16.84 | <.001 | 279.90 | 16.80 | <.001 | 230.30 | 13.93 | <.001 |
| Intercept of groups | 22.14 | 9.50 | .020 | 19.36 | 9.92 | .051 | 16.22 | 7.76 | .036 |
| Slope dose-effects |  |  |  | 0.01 | 0.01 | .433 |  |  |  |
| Model fit indices |  |  |  |  |  |  |  |  |  |
| Marginal pseudo R^2^ | 0 |  |  | .001 |  |  | .214 |  |  |
| Conditional pseudo R^2^ | .077 |  |  | .090 |  |  | .269 |  |  |
| -2 Log Likelihood | 5422.30 |  |  | 5425.56 |  |  | 5271.44 |  |  |
| BIC | 5435.20 |  |  | 5444.90 |  |  | 5284.32 |  |  |
| AIC | 5426.30 |  |  | 5431.56 |  |  | 5275.44 |  |  |

*Note.* Intraclass correlation for Model A = .077. MASC = Multidimensional Anxiety Scale for Children; GL = group leader; BIC = Bayesian information criterion; AIC = Akaike information criterion.

^a^ Reference category is the lowest value. Sex of child: girls = 0, boys = 1; Delivery format: blended = 1, group = 2; Parental involvement: low = 1, high = 2.
