# Supplementary material for: Dose-Response Effects of MittEcho, a Measurement Feedback System, in an Indicated Mental Health Intervention for Children in Municipal and School Services in Norway
Source: Adm Policy Ment Health. 2024 May 29;52(1):223–40. doi: 10.1007/s10488-024-01389-9 (PMC11703986; doi:10.1007/s10488-024-01389-9)
Supplement: Supplementary file 4 — Supplementary Material 4 [file 10488_2024_1389_MOESM4_ESM.docx]

**Supplementary 4**

**Table 10**

*Fixed, Random Effects and Model Fit Indices for Model A, B and Final Mode (C) for the Dose-Response Effect of MFS Implementation on Changes in Depression Scores*

| Effects | Model A | | | Model B | | | Model C | | |
| --- | --- | --- | --- | --- | --- | --- | --- | --- | --- |
|  | estimate | *SE* | *p* | estimate | *SE* | *p* | estimate | *SE* | *p* |
| Fixed effects |  |  |  |  |  |  |  |  |  |
| Intercept | 2.35 | 0.25 | <.001 | 2.31 | 0.28 | <.001 | −4.43 | 3.78 | .241 |
| Implementation Index |  |  |  | 3*10^−3^ | 0.01 | .794 | 2*10^−3^ | 0.01 | .877 |
| SMFQ pre intervention |  |  |  |  |  |  | 0.51 | 0.04 | <.001 |
| Sex of child ^a^ |  |  |  |  |  |  | −0.40 | 0.44 | .357 |
| Child age |  |  |  |  |  |  | −0.19 | 0.34 | .569 |
| Attendance Emotion |  |  |  |  |  |  | 0.04 | 0.01 | .002 |
| Delivery format Emotion ^a^ |  |  |  |  |  |  | −0.50 | 0.50 | .318 |
| Parental involvement ^a^ |  |  |  |  |  |  | 0.07 | 0.52 | .886 |
| GL experience |  |  |  |  |  |  | 0.01 | 0.26 | .972 |
| Random effects |  |  |  |  |  |  |  |  |  |
| Residual | 35.89 | 2.16 | <.001 | 35.82 | 2.16 | <.001 | 27.55 | 1.70 | <.001 |
| Intercept of groups | 0.86 | 1.01 | .391 | 0.56 | 1.10 | .611 | 2.14 | 1.02 | .036 |
| Slope dose-effects |  |  |  | 0.00 | 0.00 | .540 |  |  |  |
| Model fit indices |  |  |  |  |  |  |  |  |  |
| Marginal pseudo R^2^ | 0 |  |  | 0 |  |  | .222 |  |  |
| Conditional pseudo R^2^ | .029 |  |  | .032 |  |  | .283 |  |  |
| -2 Log Likelihood | 4090.58 |  |  | 4097.28 |  |  | 3947.55 |  |  |
| BIC | 4103.48 |  |  | 4116.61 |  |  | 3960.42 |  |  |
| AIC | 4094.58 |  |  | 4103.28 |  |  | 3951.55 |  |  |

*Note.* Intraclass correlation for Model A = .029. SMFQ = Mood and Feelings Questionnaire – Short version for children; GL= group leader; BIC = Bayesian information criterion; AIC = Akaike information criterion.

^a^ Reference category is the lowest value. Sex of child: girls = 0, boys = 1; Delivery format: blended = 1, group = 2; Parental involvement: low = 1, high = 2.
