# Supplementary material for: Dose-Response Effects of MittEcho, a Measurement Feedback System, in an Indicated Mental Health Intervention for Children in Municipal and School Services in Norway
Source: Adm Policy Ment Health. 2024 May 29;52(1):223–40. doi: 10.1007/s10488-024-01389-9 (PMC11703986; doi:10.1007/s10488-024-01389-9)
Supplement: Supplementary file 5 — Supplementary Material 5 [file 10488_2024_1389_MOESM5_ESM.docx]

**Supplementary 5**

**Table 11**

*Fixed Effects, Random Effects and Model Fit Indices for Model A, B and Final Model (C) for the Dose-Response Effect of MFS Implementation on User Satisfaction with the Emotion Program*

| Effects | Model A | | | Model B | | | Model C | | |
| --- | --- | --- | --- | --- | --- | --- | --- | --- | --- |
|  | estimate | *SE* | *p* | estimate | *SE* | *p* | estimate | *SE* | *p* |
| Fixed effects |  |  |  |  |  |  |  |  |  |
| Intercept | 7.64 | 0.10 | <.001 | 7.64 | 0.11 | <.001 | 10.72 | 1.46 | <.001 |
| Implementation Index |  |  |  | 0.00 | 4*10^−3^ | .940 | −1*10^−3^ | 4*10^−3^ | .863 |
| SMFQ change score |  |  |  |  |  |  | 0.04 | 0.01 | .014 |
| MASC change score |  |  |  |  |  |  | −0.00 | 0.01 | .949 |
| Sex of child ^a^ |  |  |  |  |  |  | 0.03 | 0.16 | .849 |
| Child age |  |  |  |  |  |  | −0.32 | 0.12 | .009 |
| Attendance Emotion |  |  |  |  |  |  | 0.00 | 0.01 | .496 |
| Delivery format Emotion ^a^ |  |  |  |  |  |  | −0.25 | 0.19 | .193 |
| Parental involvement ^a^ |  |  |  |  |  |  | −0.07 | 0.20 | .711 |
| GL experience |  |  |  |  |  |  | −0.10 | 0.10 | .304 |
| Random effects |  |  |  |  |  |  |  |  |  |
| Residual | 3.27 | 0.20 | <.001 | 3.28 | 0.20 | <.001 | 3.25 | 0.20 | <.001 |
| Intercept of groups | 0.48 | 0.15 | <.001 | 0.48 | 0.15 | <.001 | 0.41 | 0.14 | .003 |
| Slope of dose-effect ^b^ |  |  |  |  |  |  |  |  |  |
| Model fit indices |  |  |  |  |  |  |  |  |  |
| Marginal pseudo R^2^ | 0 |  |  | 0 |  |  | .038 |  |  |
| Conditional pseudo R^2^ | .128 |  |  | .129 |  |  | .146 |  |  |
| -2 Log Likelihood | 2596.33 |  |  | 2605.49 |  |  | 2618.57 |  |  |
| BIC | 2609.21 |  |  | 2618.37 |  |  | 2631.42 |  |  |
| AIC | 2600.33 |  |  | 2609.49 |  |  | 2622.57 |  |  |

*Note.* Intraclass correlation for Model A = .128. MASC = Multidimensional Anxiety Scale for Children; SMFQ = Mood and Feelings Questionnaire – Short version for children; GL= group leader; BIC = Bayesian information criterion; AIC = Akaike information criterion.

^a^ Reference category is the lowest value. Sex of child: girls = 0, boys = 1; Delivery format: blended = 1, group = 2; Parental involvement: low = 1, high = 2.

^b^ Effect could not be estimated.
